# Working memory in context: The role of alcohol distractors in working memory performance in low to heavy alcohol drinkers

**DOI:** 10.1111/acer.70232

**Published:** 2026-01-12

**Authors:** Karis Colyer‐Patel, Emese Kroon, Christophe Romein, Hanan El Marroun, Janna Cousijn

**Affiliations:** ^1^ Neuroscience of Addiction (NofA) Lab, Center for Substance Use and Addiction Research (CESAR), Department of Psychology, Education & Child Studies Erasmus University Rotterdam Rotterdam The Netherlands; ^2^ UMC Brain Center, Department of Psychiatry University Medical Center Utrecht Utrecht The Netherlands; ^3^ Department of Child and Adolescent Psychiatry/Psychology, Erasmus MC University Medical Center Rotterdam Rotterdam The Netherlands; ^4^ Department of Psychology, Education & Child Studies, Erasmus School of Social and Behavioural Sciences Erasmus University Rotterdam Rotterdam The Netherlands

**Keywords:** alcohol use, alcohol use disorder, attentional bias, risk factors, working memory

## Abstract

**Background:**

Motivational and cognitive control‐related processes both play a role in addiction but are often studied independently. Alcohol‐related cues may impair performance in cognitively demanding tasks, particularly in individuals with alcohol use‐related problems, where working memory (WM) may be especially affected. This study investigated whether distracting alcohol‐related flankers impact WM performance across varying levels of alcohol use severity.

**Methods:**

A total of 310 participants were classified into risk groups based on Alcohol Use Disorder Identification Test (AUDIT) scores: low (≤7), mid (8–14), and high (≥15). We developed an online N‐back flanker task where letters were flanked by alcohol‐related or neutral words. Four WM‐loads (0‐, 1‐, 2‐, 3‐back) were included, with higher loads requiring participants to hold and update more information in WM. Linear mixed effects models assessed the effects of WM‐load, flanker type, group, or their interaction on accuracy (% correct) and reaction time.

**Results:**

An interaction was found between WM‐load and flanker type; reduced accuracy (*B* = −2.47; *p*_Holm_ = 0.002) and longer reaction times (*B* = 58.46; *p*_Holm_ < 0.001) were found when participants were presented with alcohol flankers and a higher WM‐load relative to neutral flankers and a lower WM‐load. Difference scores (3‐back minus 1‐back) showed that individuals in the mid‐risk group had a larger reduction in accuracy (*B* = −4.12; *p*_Holm_ = 0.021) when presented with alcohol versus neutral flankers, relative to the low‐risk group. For reaction time, only an effect of flanker type was found, with shorter reaction times (*B* = −29.93; *p*_Holm_ = 0.012) for alcohol flankers versus neutral flankers.

**Conclusions:**

Our findings suggest that a distracting alcohol‐related context negatively impacts WM performance, particularly under high cognitive demand. This effect is particularly pronounced in mid‐risk alcohol users. This suggests that alcohol‐related cognitive interference may be more significant during the early stages of problematic alcohol use.

## Graphical abstract

Alcohol‐related distracting cues were found to impair working memory (WM) performance under high cognitive demand. Across the sample, alcohol flankers were associated with reduced accuracy and slower responses as WM load increased. Examining load‐dependent change, individuals in the mid‐risk (AUDIT 8–14) group showed a greater decline in accuracy from low to high WM load when alcohol cues were present, relative to the low‐risk (AUDIT≤7) group. This suggests that alcohol‐related cognitive interference may be more significant during early stages of problematic alcohol use.

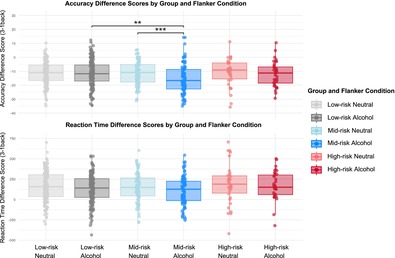

## INTRODUCTION

With an estimated 400 million individuals globally living with alcohol use disorder (AUD) and 1.81 million deaths per year attributed to alcohol consumption (Ritchie & Roser, 2022; WHO, 2024), the need to unravel the etiology of AUD remains critical. While it is widely known that both motivational and cognitive control‐related processes are important in AUD (Meyer et al., 2016; Wilcox et al., 2014), most studies have focused on these processes independently. Motivational stimuli, such as alcohol‐related cues that trigger the urge to use, may have a profound impact on performance in cognitively demanding situations (Petit et al., 2012). This effect could be especially significant in individuals with AUD, where control over these triggered urges to use is significantly compromised (Baler & Volkow, 2006). To address this, this study explored whether alcohol‐related distractors negatively impact working memory (WM) performance in individuals with varying severity of alcohol use.

Cognitive control is an umbrella term encompassing several executive functions. While exact definitions vary, it generally refers to the selection and execution of goal‐directed action in conflicting contexts, as well as the ability to inhibit prepotent responses (Mackie et al., 2013). WM is one key component of cognitive control and can be defined as the ability to maintain, process, and update information in the absence of new sensory input (Pusch et al., 2023). Findings regarding WM performance in alcohol users or individuals with AUD are mixed. Some studies report impairments in heavy drinkers (Mahedy et al., 2018; Parada et al., 2012) and individuals with AUD (Stavro et al., 2013; Wesley et al., 2017), while others find no differences in performance between individuals with AUD and controls (Quaglino et al., 2015; Tapert et al., 2004; Vollstädt‐Klein et al., 2010). These discrepancies may partly depend on the type of WM task performed. For example, performance on a verbal task did not differ between individuals with AUD and controls, whereas spatial WM of the AUD group was disproportionately impaired on recall following an arithmetic distractor task (Chanraud et al., 2010). Longitudinal studies further highlight the complexity of the association between alcohol use and WM. For example, binge‐drinking adolescents who continue to binge in early adulthood show reduced WM performance compared to nonbinge drinkers, emphasizing the need to consider patterns of alcohol use over time (Carbia et al., 2017).

In parallel, heightened reactivity to alcohol‐related cues, termed cue‐reactivity, is a well‐established behavioral characteristic of AUD (e.g., Schacht et al., 2013). According to the incentive salience model (Robinson & Berridge, 1993), repeated pairing of cues with alcohol use leads these cues to acquire motivational significance, such that they can trigger conditioned responses including craving and physiological arousal (Ray & Roche, 2018; Zeng et al., 2021). These alcohol‐related cues tend to capture attention more than neutral cues, a phenomenon known as attentional bias, which is typically more pronounced in individuals with heavy drinking patterns or AUD and has been linked to increased alcohol use and relapse (Bollen et al., 2022; Cox et al., 2006). Importantly, a body of research indicates that cue‐reactivity and attentional bias can interfere with cognitive control processes, including working memory (Wilcox et al., 2014).

For instance, individuals with AUD have been shown to have impaired response inhibition, especially in the presence of alcohol cues. For example, during the Alcohol‐Shifting task, they had longer reaction times (RT) and more commission errors when exposed to alcohol‐related versus neutral words (Noël et al., 2007). Similarly, heavy social drinkers made more errors than light drinkers, but only when alcohol‐related backgrounds were used as task‐irrelevant distractors (Petit et al., 2012). In adapted cued go/no‐go tasks, adult drinkers showed greater inhibitory failures when alcohol images were presented, a pattern that was replicated under acute intoxication and linked to higher self‐reported alcohol consumption (Weafer & Fillmore, 2012, 2015). Alcohol‐related interference was also seen in individuals with AUD and Korsakoff syndrome during an alcohol‐adapted flanker task, though deficits varied depending on task complexity (Brion et al., 2018). Even among nondependent social drinkers, slower RT were found on trials with an alcohol‐related background, particularly under high cognitive loads, and this effect scaled with weekly alcohol intake (Nikolaou et al., 2013). Together, these findings suggest that distracting alcohol‐related cues may impair cognitive control‐related processes in individuals with AUD but also in heavy drinkers. This highlights the importance of investigating bias across differing levels of AUD risk to better understand the trajectory toward the disorder.

To our knowledge, no studies have yet examined how alcohol‐related contexts or cues influence WM performance in alcohol users. However, in one study from our group, we added cannabis‐related or neutral flankers to a n‐back WM task (Kroon et al., 2022). Unlike, Stroop‐type tasks, these flankers did not map onto task responses, allowing us to examine the interference due to attentional capture rather than response conflict. Participants had to selectively attend to the central letter and ignore the distracting flankers. According to load theory of selective attention (De Fockert, 2013; Lavie et al., 2004), active control against processing distractors requires available WM resources. When WM capacity is heavily loaded, fewer resources remain for suppressing distractors, leading to increased interference from these irrelevant stimuli. Moreover, the salience of distractors also appears to impact the ability to maintain attentional control under higher WM loads. For example, Carmel et al. (2012) found that irrelevant faces presented as task distractors were more likely to be identified when concurrent WM load was high, but this was not found for less salient stimuli (buildings; Carmel et al., 2012). In the context of our study, alcohol cues that are highly salient for alcohol users may similarly capture attention and reduce WM performance. To date, no studies have directly investigated this in alcohol users, leaving open the question of whether WM is generally susceptible to interference from salient alcohol distractor processes (De Fockert & Bremner, 2011).

Hence, this study aimed to investigate whether task‐irrelevant alcohol‐related contextual cues negatively impact WM performance (accuracy and RT) in individuals with low to high alcohol use severity. We developed a novel online N‐back alcohol flanker task based on a previous version developed for cannabis (Kroon et al., 2022). We hypothesized that more severe alcohol use would be associated with poorer WM, and that this group effect would be more prominent when individuals were presented with alcohol flankers relative to neutral flankers.

## MATERIALS AND METHODS

This study was part of a larger online project aiming to investigate associations between social reward sensitivity, cognitive control, mental well‐being, and alcohol and cannabis use across the lifespan (Kroon et al., 2024). Only those who completed the N‐back alcohol flanker task were included in this study. Study protocols were approved by the Ethics Review Committee of Erasmus University Rotterdam (ETH2122‐0311), and all participants provided informed consent before participation. After the completion of the study, all participants were entered into a raffle, whereby one in five participants received a €10 gift voucher, and four participants received a €50 prepaid credit card.

### Participants and procedure

A total of 310 participants aged 16–80 completed the N‐back alcohol flanker task. Participants were recruited via in‐person flyers (Amsterdam and Rotterdam region), social media (Instagram, Facebook, and WhatsApp), and the online research platform Prolific. Advertisement focused on individuals who use alcohol and/or cannabis. Eligibility criteria required participants to be 16 years or above and to be proficient in either English or Dutch, the study languages. The online study lasted approximately 1 h and included several questionnaires, followed by three experimental tasks and concluded with a final set of questionnaires.

### Measures

#### N‐back alcohol flanker task

Participants completed an adapted version of the standard letter N‐back task, developed using the Gorilla Experiment builder (Anwyl‐Irvine et al., 2020). Each letter presented in the middle of the screen was flanked bilaterally by either alcohol‐related words (i.e., beer, wine) or neutral office‐related words (i.e., pen, lamp). The selected words had been previously validated within our group (Cousijn et al., 2015) and were matched for length and syllable count. The full list of alcohol‐related and neutral flanker words is provided in the supplementary material (Data S1)

**FIGURE 1:**
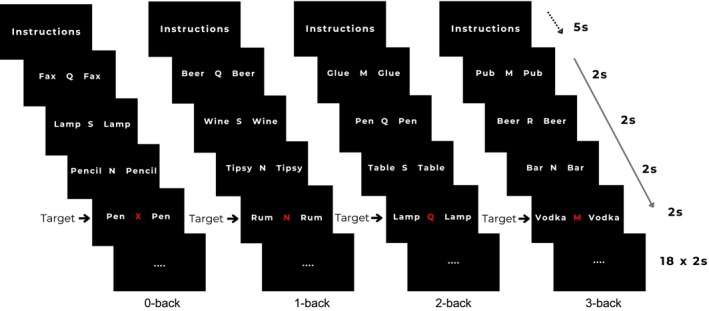
Overview of the N‐back flanker task. Instructions were provided at the beginning of the task, and at the start of each block, a brief block‐specific instruction screen was displayed for 5 s. Each block consisted of 18 trials, with each trial lasting 2 s, leading to a total block duration of 36 s. During each trial, a letter appeared in the centre of the screen, with words displayed on both sides for the entire trial. These flanking words were either neutral or alcohol‐related. The target letters in the examples shown are colored red.

The task included four WM‐loads: 0‐back (recognition), 1‐back, 2‐back, and 3‐back, with higher N‐back levels requiring participants to hold and update more information in WM. In total, there were eight conditions, combining two flanker types (alcohol and neutral) with four WM‐load levels (0‐back, 1‐back, 2‐back, 3‐back). Each condition was presented twice, resulting in 16 blocks. Blocks were presented in a fixed pseudorandomized order, identical for all participants, such that alcohol and neutral blocks were interleaved and no two blocks of the same condition occurred consecutively. Each block began with a five‐second instruction screen, which also served as a brief break, followed by 18 letter trials (five targets, 13 nontargets) in which each letter and their bilateral flankers were presented for 2 s (Figure 1). During the 0‐back, participants were instructed to press the target button when the letter “X” appeared. For the 1‐back, 2‐back, and 3‐back, participants pressed the target button when the presented letter was identical to the letter shown one, two, or three trials earlier, respectively. For the nontarget trials, participants were instructed to press the nontarget button. Each condition contained an equal number of targets and nontargets when summed across its two blocks. Accuracy was calculated as the proportion of correct responses across both target and nontarget trials, and RT was defined as the mean latency for correct responses. The entire task lasted 11 minutes, and no feedback on performance was provided.

#### Questionnaires

Participants completed demographic questions, including age, sex, nationality, and highest completed level of education. Due to the differences in education systems across countries, education levels were recoded to allow for comparisons: (1—less than high school education, 2—(pre)vocational or college, 3—(pre)university). Additionally, participants completed questionnaires related to their history of alcohol use, including age of onset, binge drinking frequency (≥5 drinks on a single occasion; Courtney & Polich, 2009), and the Alcohol Use Disorder Identification Test (AUDIT; Saunders et al., 1993) to assess alcohol consumption and related problems. The primary outcome measure for alcohol use was the AUDIT. Furthermore, participants completed the Cannabis Use Disorder Identification Test‐Revised (CUDIT‐R; Adamson et al., 2010) to assess cannabis use and related problems. Additionally, substance use of the past 2 weeks was assessed using the Timeline Follow‐Back (TLFB) method (Robinson et al., 2014), in which participants indicated the frequency and quantity of use over the last 14 days for alcohol, cannabis, and tobacco. The TLFB started from the day before testing; therefore, participants' substance use on the actual day of task completion was not assessed. Participants were also not specifically asked to abstain from alcohol, cannabis, or other substances on the day of testing, which is common in remote, online research designs. Participants also provided an estimate of their lifetime drug use (excluding alcohol, cannabis, and tobacco).

### Data analyses

To investigate the differential effect of distracting alcohol flankers on WM across differing levels of AUD risk, participants were classified into three groups based on their AUDIT score: low (AUDIT ≤7; *n* = 181), mid (AUDIT 8–14; *n* = 93), and high (AUDIT ≥15; *n* = 36). A score of 7 or below is considered indicative of low‐risk alcohol consumption, while a score of 8 or more suggests hazardous drinking, and a score of 15 or more indicates likely alcohol dependence (Saunders et al., 1993). While the smallest group was underpowered to detect small effects (*f* = 0.1), the overall design had sufficient power (power >0.96) to detect large effects (*f* = 0.4) across all groups. ANOVAs, or nonparametric alternative Kruskal–Wallis tests, were used to compare groups on all included continuous variables, depending on whether parametric assumptions were met. Chi‐squared tests or Fisher's exact tests in case of small sample sizes (*n* ≤5) were used to compare groups on categorical variables. Post hoc pairwise comparisons were conducted to further investigate any significant effects.

N‐back alcohol flanker task trials without a response or with a RT below 200 ms were excluded to remove implausibly fast responses that may indicate premature or accidental key presses (*N* of trials excluded = 4121). Additionally, blocks with an average accuracy below 60% were excluded (N of blocks excluded = 182). Given the novelty of the task, we took a two‐step approach. The first step aimed to provide a comprehensive overview of performance across all the task conditions. The second step focused on a more targeted and statistically powered analysis, concentrating on key comparisons most relevant to our hypotheses.

First, we used a linear mixed effects model approach with maximum likelihood estimation, specifying random intercepts for participants and random slopes for WM‐load and flanker type to account for within subjects repeated measures (Meteyard & Davies, 2020). This allowed us to examine the effects of WM‐load, flanker type, group, and their interactions on task performance. Separate models were run using accuracy (percentage of correct responses) and reaction time (RT) on accurate trials as the outcome to assess whether the task performed as expected. The model with the best fit was selected using a stepwise, nested model comparison approach, in which predictors were added sequentially. We began with a baseline model and then added WM‐load, flanker type, and their interaction (WM‐load × Flanker type), followed by group, the interactions with WM‐load and flanker type, and finally the three‐way interaction (WM‐load × Flanker type × Group). This procedure resulted in eight models in total. Model fit was then evaluated using Akaike's Information Criterion (AIC), which favors predictive accuracy (Vrieze, 2012), and lower values indicate a better fit.

Second, we reduced model complexity by subtracting performance (accuracy and RT) on the low‐load (1‐back) condition from the high‐load (3‐back) condition for both alcohol and neutral flanker trials. This provided a more targeted index of cognitive control under high load, aligning with the behavioral and neural 3‐back versus 1‐back contrasts often used in N‐back studies (Dima et al., 2014; Ravizza et al., 2004; Schmidt et al., 2009). A linear mixed effects model with maximum likelihood estimation and random intercept was conducted to examine the effects of flanker type, group, and their interaction on these WM difference scores. Once more, the model with the best fit was selected using a stepwise approach, starting with a baseline model, followed by the addition of flanker type, group, and their interaction (Flanker type × Group). This procedure resulted in four models in total. Given that sex, age, and other substance use differed between groups, and considering emerging group effects, we conducted a series of sensitivity analyses by including each as a covariate in turn, followed by a model that included all covariates simultaneously to assess whether our findings remained robust. In addition, to ensure that findings were not driven by older participants, we repeated the analyses after excluding individuals over 65. To account for multiple testing across fixed effects in our model comparisons, Holm's correction was first applied to all *p*‐values extracted from the full set of models. For effects that remained significant after this correction, post hoc pairwise comparisons were conducted. Holm's correction was again applied within each set of post hoc comparisons to control for multiple testing. All analyses were conducted in RStudio (version 224.4.2.764) using R (version 4.2.3; R Core Team, 2023).

To evaluate the reliability of the novel N‐back alcohol flanker task, we focused on RT, as accuracy is typically affected by learning effects across repeated blocks, which can distort reliability estimates. Task performance was visualized to inspect expected patterns across blocks, such as practice effects and fatigue. We then assessed internal consistency both within and across WM load conditions per block using Cronbach's *α*, following recommendations for reporting task reliability (Pennington et al., 2021). Reliability analyses were performed using the *psych* package in R (Revelle, 2007).

## RESULTS

### Sample characteristics

Groups did not significantly differ on education history (*p* = 0.565) or nationality (*p* = 0.522). However, the high‐risk group included significantly more males (*p* = 0.014) and was younger (*p* = 0.004) compared to the low‐risk group (see Table 1). The mid‐risk group was also younger (*p* = 0.003) than the low‐risk group.

**TABLE 1:** Sample characteristics.

Measures	Total, *n* = 310	Low risk (AUDIT ≤7), *n* = 181	Mid risk (AUDIT 8–14), *n* = 93	High risk (AUDIT ≥15), *n* = 36	Test statistic and *p*‐value	Significant group differences (*p* values)
Age, mean (SD)	34.53 (14.71)	37.20 (14.73)	30.80 (13.99)	30.78 (13.82)	*χ* ^2^(2) = 20.95, *p* < 0.001	↑ Age of Low vs. High (*p* = 0.009) ↑ Age of Low vs. Mid (*p* = 0.003)
Sex, % male/female/other	51.0/48.1/1.0	42.5/56.4/1.1	50.5/48.4/1.1	69.4/30.6/0.0	Fisher's Exact Test *p* = 0.008	↑ Proportion of males in High vs. Low (*p* = 0.014)
Nationality, % Dutch	31.0	29.3%	35.5	27.8	*χ* ^2^(2) = 1.30, *p* = 0.522	ns
Education level, % less than high school, (pre)vocational or college, (pre)university	1.9/ 14.2/79.7	1.15/15.5/83.3	3.4/12.4/84.3	2.9/17.7/79.4	Fisher's Exact Test *p* = 0.565	ns
AUDIT, mean (SD)	7.73 (5.62)	3.97 (2.00)	10.60 (1.81)	19.10 (4.58)	*χ* ^2^(2) = 239.79, *p* < 0.001	↑ score in Mid vs. Low (*p* < 0.001) ↑ score in High vs. Low (*p* < 0.001) ↑ score in High vs. Mid (*p* < 0.001)
Alcohol use age of onset, mean (SD)	15.09 (3.08)	15.49 (3.17)	14.70 (2.84)	14.08 (2.96)	*χ* ^2^(2) = 11.704, *p* = 0.003	↑ Age of onset in Low vs. High (*p* = 0.012) ↑ Age of onset in Low vs. Mid (*p* = 0.004)
Binge drinking days last year, mean (SD)	26.50 (52.51)	8.22 (13.41)	33.90 (43.37)	99.69 (105.08)	*χ* ^2^(2) = 112.96, *p* < 0.001	↑ Binge days in High vs. Low (*p* < 0.001) ↑ Binge days in High vs. Mid (*p* < 0.001) ↑ Binge days in Mid vs. Low (*p* < 0.001)
Last 14‐day alcohol use (TLFB – standard drinks), mean (SD)	17.34 (22.13)	8.26 (11.13)	23.31 (18.84)	47.63 (35.88)	*χ* ^2^(2) = 106.49, *p* < 0.001	↑ Alcohol use in High vs. Low (*p* < 0.001) ↑ Alcohol use in High vs. Mid (*p* = 0.003) ↑ Alcohol use in Mid vs. Low (*p* < 0.001)
CUDIT‐R, mean (SD)	3.81 (6.55)	2.36 (5.10)	6.04 (7.91)	5.28 (7.37)	*χ* ^2^(2) = 27.50, *p* < 0.001	↑ score in High vs. Low (*p* = 0.003) ↑ score in Mid vs. Low (*p* < 0.001)
Last 14‐day cannabis use (TLFB ‐ grams), mean (SD)	1.12 (3.40)	0.66 (2.28)	1.79 (4.69)	1.74 (3.83)	*χ* ^2^(2) = 15.53, *p* < 0.001	↑ Cannabis use in High vs. Low (*p* = 0.002) ↑ Cannabis use in Mid vs. Low (*p* < 0.001)
Tobacco Daily use, %	21.3	14.9	22.6	50.0	*χ* ^2^(2) = 22.19, *p* < 0.001	↑ Daily smokers in High vs. Low (*p* < 0.001) ↑ Daily smokers in High vs. Mid (*p* = 0.005)
Last 14‐day cigarette use (TLFB – cigarettes), mean (SD)	34.40 (84.56)	22.22 (71.94)	38.63 (88.30)	84.72 (112.30)	*χ* ^2^(2) = 24.706, *p* < 0.001	↑ Cannabis use in High vs. Low (*p* < 0.001) ↑ Cannabis use in High vs. Mid (*p* = 0.014) ↑ Cannabis use in Mid vs. Low (*p* = 0.001)
Other lifetime drug use^a^, %	10.3	4.4	19.4	16.7	Fischer's Exact Test *p* < 0.001	↑ Ever used other drugs in High vs. Low (*p* = 0.001) ↑ Ever used other drugs in Mid vs. Low (*p* = 0.001)

Abbreviations: AUDIT, Alcohol Use Disorder Identification Test; CUDIT‐R, Cannabis Use Disorder Identification Test; ns, not significant; SD, standard deviation; TLFB, timeline follow‐back

^a^ Including all drugs except alcohol, tobacco and cannabis. Binge drinking information was available for 308 individuals for the whole group, and 91 individuals in the mid‐risk group.

The high‐risk group reported more binge drinking days and greater alcohol consumption in the past 14 days than both the mid‐ and low‐risk groups (all *p* ≤ 0.003) and started drinking earlier than the low‐risk group (*p* = 0.012). The mid‐risk group also started drinking earlier than the low‐risk group (*p* = 0.004) and exceeded the low‐risk group on the other two measures (all *p* ≤ 0.001).

Alcohol‐using groups also differed on other substance‐related variables. The high‐risk group reported higher past 14‐day cigarette use (all *p* = 0.014) and had a greater proportion of daily smokers (all *p* ≤ 0.005) than other groups. Additionally, past 14‐day cigarette use was higher in the mid‐risk compared to the low‐risk group (*p* = 0.001). Cannabis use in the past 14 days was higher in the high and mid‐risk groups compared to the low‐risk group (all *p* ≤ 0.002). Lifetime use of other drugs (excluding cannabis, alcohol, and tobacco) was also more common in the mid‐risk and high‐risk groups than in the low‐risk group (all *p =* ≤ 0.001).

### N‐back performance

#### Accuracy

**FIGURE 2:**
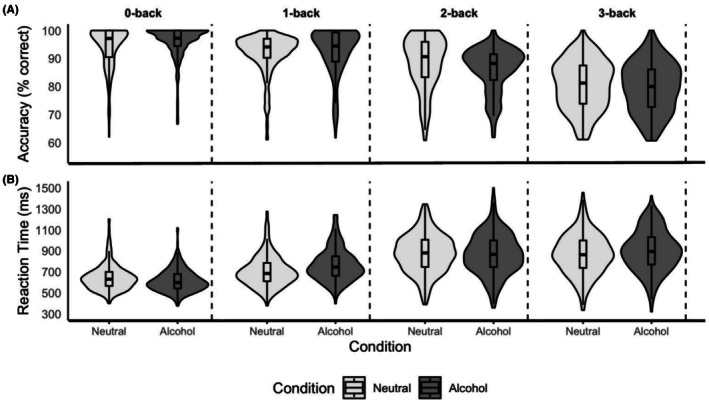
N‐back alcohol flanker task performance. (A) Accuracy for 0‐back, 1‐back, 2‐back and 3‐back across flanker type. Boxplots overlaid show the median and interquartile range. (B) Reaction time for 0‐back, 1‐back, 2‐back and 3‐back across flanker type. Boxplots overlaid show the median and interquartile range.

For accuracy, a significant main effect of WM‐load was found: as load increased, accuracy reduced, suggesting that the task worked as intended. A significant interaction between WM‐load and flanker type was also found, suggesting that the effect of WM‐load on accuracy varied depending on the flanker type. For both the 2‐back and 3‐back (not the 1‐back) compared to the 0‐back, accuracy was lower for trials with alcohol flankers compared to the neutral flankers (Table 2 and Figure 2). Across all accuracy models, no group effects or interactions with group were observed: the effects of WM and flanker type were similar across groups (see Table S1 for model selection).

**TABLE 2:** Fixed and random effects for the best‐fitting interaction model (WM‐load × flanker) predicting accuracy and reaction time.

Model	Model coefficients					
	Fixed effects			Random effects		
	*B*	95% CI (*B*)	SE (*B*)	*t*	*p* *	SD
Accuracy						
(Intercept)	94.06	92.14: 94.98	0.47	200.75	<0.001	5.29
WM‐load: 1‐back	−2.75	−3.75: −1.75	0.51	−5.38	<0.001	3.69
WM‐load: 2‐back	−5.99	−7.00: −4.98	0.51	−11.68	<0.001	
WM‐load: 3‐back	−13.98	−14.99: −12.97	0.52	−27.14	<0.001	
Flanker type: alcohol	1.73	0.92: 2.55	0.42	4.16	0.002	4.68
WM‐load 1‐back: flanker alcohol	−0.89	−2.05: 0.26	0.59	−1.51	1.0	
WM‐load 2‐back: flanker alcohol	−4.34	−5.51: −3.18	0.59	−7.31	<0.001	
WM‐load 3‐back: flanker alcohol	−2.47	−3.63: −1.30	0.60	−4.15	0.002	
Reaction time						
(Intercept)	645.83	627.41: 664.24	9.40	68.73	<0.001	118.19
WM‐load: 1‐back	61.07	43.87: 80.42	9.32	6.55	<0.001	85.78
WM‐load: 2‐back	234.21	215.93: 252.48	9.31	25.14	<0.001	
WM‐load: 3‐back	222.98	204.69: 241.27	9.32	23.92	<0.001	
Flanker type: alcohol	−27.37	−39.62: −15.13	6.23	−4.40	<0.001	68.53
WM‐load 1‐back: flanker alcohol	87.55	70.13: 104.81	8.83	10.03	<0.001	‐
WM‐load 2‐back: flanker alcohol	18.24	0.87: 35.62	8.84	2.06	1.0	‐
WM‐load 3‐back: flanker alcohol	58.46	41.09: 75.83	8.83	6.62	<0.001	

*Note*: Mixed model results using random intercept and maximum likelihood estimation. Baseline references were the 0‐back condition and Neutral flankers.

Abbreviations: CI, confidence interval; SD, standard deviation; SE, standard error; WM, working memory.

* Holm corrected *p*‐values are displayed.

#### Reaction time

For RT, a significant main effect of WM‐load was found, with RT increasing as load increased. A significant interaction between WM‐load and flanker type was also found. When comparing both the 3‐back and 1‐back (but not the 2‐back) to the 0‐back, RT was longer for the alcohol compared to neutral flankers (Table 2 and Figure 2). Across all RT models, no group effects or interactions with group were observed (see Table S2 for model selection).

### Flanker effect on difference score (3‐back–1‐back)

#### Accuracy

**FIGURE 3:**
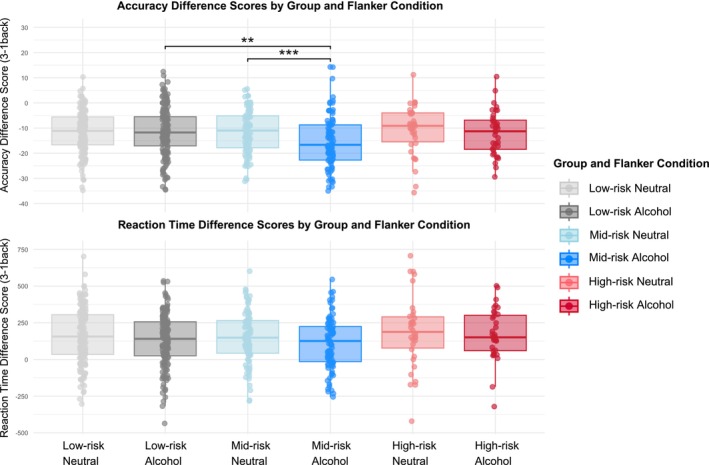
Accuracy and difference score across groups and flanker type. The middle line inside the box represents the median, and the line shows the interquartile range. Holm corrected *p*‐values: **p < 0.01; ***p < 0.001.

For accuracy, the best‐fitting model included a significant interaction between flanker type and group (see Table 3; see Table S3 for model selection), whereby individuals in the mid‐risk group showed a larger reduction in accuracy from the 1‐back to the 3‐back condition when presented with alcohol flankers compared to neural flankers relative to the low‐risk group. This effect remained significant when controlling for sex, age, and illicit substance use. In contrast, in the high‐risk group, flanker type did not differentially affect accuracy across the 1‐back and 3‐back conditions when compared to the low‐risk group (Figure 3).

**TABLE 3:** Model fit statistics for best fitting model. For accuracy this was the interaction model (flanker × group) controlling, and for reaction time this was the flanker model.

Model	Model coefficients					
	Fixed effects			Random effects		
	*B*	95% CI (*B*)	SE (*B*)	*t*	*p* *	SD
Difference score of accuracy (3‐back – 1‐back)						
(Intercept)	−11.31	−12.72: −9.90	0.72	−15.71	<0.001	5.86
Flanker type: alcohol	−0.11	−1.64: 1.41	0.78	−0.15	1.0	6.41
Group mid risk	0.05	−2.34: 2.44	1.22	0.04	1.0	
Group high risk	0.37	−3.04: 3.78	1.74	0.21	1.0	
Flanker alcohol: group mid risk	−4.12	−6.74: −1.50	1.34	−3.08	0.021	
Flanker alcohol: group high risk	−0.89	−4.54: 2.77	1.86	−0.47	1.0	
Difference score of reaction time (3‐back – 1‐back)						
(Intercept)	161.04	140.62: 181.46	10.39	15.49	< 0.001	140.42
Flanker type: alcohol	−29.93	−48.15: −11.72	9.27	−3.23	0.012	102.63

*Note*: Mixed model results using random intercept and maximum likelihood estimation. Baseline references were Neutral flankers and the low risk group.

Abbreviations: CI, confidence interval; SD, standard deviation; SE, standard error.

* Holm corrected *p*‐values are displayed. Sensitivity analyses excluding participants aged 65+ showed that the group × flanker type effect remained significant (*B* = −4.31, SE = 1.39, *t*(257) = 3.10, *p*_Holm_ <0.02).

To further explore this interaction, we examined within‐ and between‐group differences. Within the mid‐risk group, participants showed a significantly larger accuracy decline on the 3‐back compared to the 1‐back when presented with alcohol flankers compared to neutral flankers (*B* = −4.23, SE = 1.09, *t*(272) = 3.89, *p*_Holm_ <0.001). No other within‐group comparisons were significant. Additionally, when investigating between‐group differences, when presented with alcohol flankers, the mid‐risk group showed significantly worse accuracy than the low‐risk group on the 3‐back compared to 1‐back (*B* = −4.07, SE = 1.24, *t*(272) = −3.29, *p*_Holm_ = 0.001).

#### Reaction time

For RT, only a main effect of flanker type was observed: alcohol flankers compared to neutral flankers were associated with a significantly smaller increase in RT from the 1‐back to the 3‐back condition (see Table 3; see Table S4 for model selection).

Given the novelty of the task, we examined its internal consistency. As expected, performance showed evidence of learning, with slightly faster RT in Block 2 relative to block 1 (Block 1 *M* = 759.27 ms; Block 2 *M* = 802.89 ms; See Figure S1). Despite this, internal consistency estimates for RT were good across blocks (*α* = 0.84 for both Block 1 and Block 2) and acceptable to good across individual working memory loads (*α* = 0.68–0.80; See Table S5).

## DISCUSSION

The current study investigated whether alcohol‐related contextual cues negatively impact WM performance in individuals with varying levels of alcohol use severity, using a novel online n‐back alcohol flanker task. The impact of alcohol‐related flankers varied across WM‐load levels, with alcohol‐related flankers predominantly associated with slower RT and reduced accuracy, especially as working‐memory load increased, regardless of risk group. This supports the task's validity and sensitivity to alcohol‐related contextual interference under increasing cognitive demand.

The N‐back task is a well‐established paradigm for measuring WM performance. Consistent with prior lab‐based studies, the current online study found that increasing WM‐load was associated with reduced accuracy and slower RT (Miller et al., 2009; Schmidt et al., 2009), aligning with the robust findings that WM performance declines as cognitive demand increases. No overall group differences in WM performance were observed across levels of alcohol use risk, consistent with some prior studies including heavy drinkers or individuals with an AUD (Quaglino et al., 2015; Tapert et al., 2004; Vollstädt‐Klein et al., 2010), though other studies report reduced WM performance in individuals with AUD (Wesley et al., 2017; See meta‐analysis by Stavro et al., 2013). These inconsistencies may reflect methodological and sample differences across studies, as the current study lacked a group of individuals with AUD. Notably, the good internal consistency of RT across blocks (Cronbach's *α* = 0.84), and within WM loads (Cronbach's *α* = 0.68–0.84), supports the reliability of this novel task, suggesting that the absence of overall group differences is unlikely to reflect measurement noise or instability. When considering the interaction between WM‐load and flanker type, accuracy declined and RT increased at the highest WM‐load (i.e., 3‐back vs. 0‐back) when alcohol flankers were presented, regardless of group. This likely reflects the high salience of the alcohol stimuli, with participants under high cognitive demand showing increased susceptibility to interference. It is also possible that this general effect reflects the inherent salience of alcohol‐related words rather than being entirely driven by participants' drinking history or cue‐specific motivational relevance.

Given the novelty of our task and the existing debate regarding whether raw data versus difference scores more accurately reflect cognitive performance (Rouder et al., 2023), we investigated both. To isolate load‐dependent WM performance, we calculated a difference score between high WM‐load (3‐back) and low WM‐load (1‐back). Interestingly, for the mid‐risk versus the low‐risk group, the decline in accuracy from the 1‐back to the 3‐back was more pronounced when alcohol cues were present, whereas no differences were observed between the mid‐ and high‐risk groups. This effect was not visible in the raw RT and accuracy data. This suggests that under conditions of increasing WM demand (i.e., 3‐back vs. 1‐back, rather than looking at general cognitive demand with 3‐back vs. 0‐back), salient alcohol‐related stimuli may more strongly disrupt WM performance in individuals who engage in hazardous drinking that do not, however, meet criteria for an AUD. Reaction times showed a main effect of flanker type, with slower increases from the 1‐back to the 3‐back for alcohol compared to neutral flankers, but this did not differ across groups. The attenuated slowing may reflect longer baseline RT during the 1‐back, indicating that response efficiency was preserved regardless of drinking risk, whereas accuracy declined more steeply in the mid‐risk group in the presence of alcohol cues.

These findings align partially with previous work showing that alcohol‐related cues can interfere with cognitive control, though prior studies typically used tasks measuring other cognitive processes (Brion et al., 2018; Noël et al., 2007; Petit et al., 2012; Weafer & Fillmore, 2012, 2015). For example, Nikolaou et al. (2013) found that nondependent social drinkers had slower responses on a flanker task with distracting alcohol backgrounds, under high cognitive load, though no accuracy effects were observed. Taken together, these studies suggest that alcohol‐related stimuli can disrupt multiple cognitive processes, producing slower responses, more errors, or reduced accuracy depending on task demands and sample characteristics. Importantly, the convergence of findings across tasks targeting different cognitive domains suggests that the impact of alcohol‐related distractors is likely a domain‐general phenomenon (De Fockert & Bremner, 2011), broadly impairing cognitive processing under high cognitive demand. According to incentive sensitization theory (Robinson & Berridge, 1993), drug‐paired cues acquire motivational salience that capture attention and divert cognitive resources, potentially explaining the reduction in cognitive performance as attention shifts from task‐relevant to motivationally salient information.

Extending this work, our findings suggest that alcohol‐related cues can specifically impair WM performance during high cognitive demand in individuals at risk of problematic alcohol use. This effect may arise because mid‐risk individuals engage in more goal‐directed drinking, rendering alcohol cues particularly salient, and these cues may grab attention and compromise cognitive control as individuals actively process these stimuli (Everitt & Robbins, 2005). In contrast, in individuals with AUD, drinking behaviors may become more habitual so that alcohol‐related cues may lose their disruptive impact as processing becomes more automated (Robinson & Berridge, 2003). Indeed, individuals with AUD have been found to exhibit a blunted subjective response to alcohol and a dissociation between positive reinforcement and craving compared to heavy drinkers (Bujarski & Ray, 2014). In line with this, alcohol‐related attentional bias in severe AUD appears to be determined by subjective craving, not cognitive load (Bollen et al., 2024). From a load theory perspective (Lavie et al., 2004), high cognitive demand reduces distractor filtering for all participants, but only in mid‐risk drinkers do alcohol cues retain sufficient motivational salience to divert attention from the task. By contrast, for high‐risk drinkers, cues may still be processed, but their reduced motivational values mean attention remains more anchored to the task. Although speculative, this interpretation suggests that alcohol cue interference may emerge most strongly at intermediate stages of problematic drinking, before motivational salience is diminished by habitual patterns of use. Given the cross‐sectional nature of our design, we cannot determine causality; mid‐risk individuals may have reduced inhibitory control under high cognitive demand, or alcohol cues may temporarily compromise their control. Future longitudinal studies are needed to determine whether cue‐related WM impairment predicts progression to more problematic drinking. Replication in larger samples, particularly including more heavy drinkers, is essential to replicate and extend these findings. Nevertheless, the current findings highlight the mid‐risk group, often overlooked in research, who may show early cognitive susceptibility before more severe or habitual patterns of use emerge.

Together, these interpretations suggest that in mid‐risk drinkers, alcohol cues retain motivational salience to capture attention, particularly when cognitive resources are taxed. This may also explain why the effect emerged in accuracy rather than RT. Unlike the Stroop or go/no‐go paradigms, the alcohol flankers in our task did not map onto task‐relevant responses. Instead, the cues likely interfered with attentional selection of the central WM stimuli, particularly during the 3‐back condition. In this sense, the task may be tapping attentional selection processes that become especially vulnerable when WM capacity is limited. This interpretation aligns with load theory of attentional control (Lavie & De Fockert, 2005), which predicts that under high WM demands, salient but task‐irrelevant stimuli are more likely to intrude on processing. In this way, alcohol distractors may compromise WM accuracy by diverting attention without necessarily slowing motor execution. Given the observed deficits in WM performance under high cognitive load in the presence of alcohol‐related cues, these findings may have important implications for at‐risk drinkers. Specifically, they suggest that the interaction between cognitive control and attentional bias may be a meaningful target for prevention efforts and interventions to reduce problematic use. A range of cognitive training paradigms have aimed to enhance cognitive processes implicated in AUD (see review by Nixon & Lewis, 2019), including WM training and cognitive bias modification approaches. While these have shown mixed success in reducing alcohol use, combining them may be more effective, simultaneously strengthening cognitive control and weakening alcohol‐related associations. Although early attempts in this direction have had limited efficacy, they offer a foundation for future development (Jones et al., 2018).

The novelty in the current study lies in its comparison across low‐, mid‐, and high‐risk drinkers, revealing that mid‐risk individuals may be particularly vulnerable to the disruptive effects of alcohol cues on WM performance, but only under high cognitive demand. This supports the idea that heavy drinkers may be especially susceptible to alcohol‐related interference. In previous studies, it was suggested that a ceiling effect may have masked potential differences (Kroon et al., 2022) and so one adaptation in the current task design was the inclusion of an additional WM‐load (3‐back), which appears to have allowed for greater differentiation across groups. Indeed, we found group differences in accuracy decline from 1‐back to 3‐back. Despite these important findings, several limitations should be acknowledged. While this study examined group differences, the high‐risk group was relatively small. Although we controlled for sex, age, and illicit drug use in our analyses, it will be important to replicate these findings in larger, more balanced samples to more fully evaluate potential age‐ and sex‐specific effects (Fama et al., 2020; Kuhns et al., 2022). Additionally, the low‐risk group had a higher mean age than the other two groups, raising the question that their drinking patterns may have been heavier earlier in life. While we observed a greater distraction effect of alcohol‐related stimuli on WM performance, we cannot rule out the possibility that this effect reflects a general heightened response to salient stimuli more generally rather than alcohol‐specific interference. In particular, because the neutral words in our task were not appetitive in nature, the observed distraction effect in mid‐risk drinkers may reflect sensitivity to appetitive or emotional stimuli more broadly. Therefore, future studies should incorporate both non‐drug appetitive and emotional stimuli as controls to better isolate the specificity of distracting alcohol‐related cues and contexts from general attentional capture. Additionally, incorporating an independent measure of attentional bias would help clarify how this cognitive process is generally affected in these groups. As this was the first use of this task design, replication is necessary to establish reliability. Finally, participants were not asked to refrain from substance use on the day of participation, and drug use on the day was not assessed. As such, potential (sub)acute effects of alcohol or other substances cannot be ruled out, which is a common limitation of online studies.

To conclude, using a novel online n‐back alcohol flanker task, our findings provide evidence that distracting alcohol‐related contextual cues negatively impact WM performance when cognitive demand is high, regardless of drinking severity. However, these effects were larger in mid‐risk alcohol users relative to low‐risk alcohol users, but not high‐risk alcohol users. This suggests that the capacity of alcohol cues and contexts to influence cognitive performance may be more pronounced during the earlier stages of heavy alcohol use. These findings highlight the utility of contextual cues, such as alcohol flankers, for revealing subtle WM deficits that may not appear in traditional tasks, and underscore how alcohol cues may disrupt cognitive control, potentially contributing to vulnerability for escalating use and AUD. Future longitudinal studies are needed to investigate the predictive value of this effect for escalation of use and the risk of developing AUD.

## CONFLICT OF INTEREST STATEMENT

The authors declare no conflicts of interest.

## Supporting information


Tables S1–S5

## Data Availability

The data that support the findings of this study are available upon request from the corresponding author. The data are not publicly available due to privacy or ethical restrictions.
