# Supplementary material for: Working memory in context: The role of alcohol distractors in working memory performance in low to heavy alcohol drinkers
Source: Alcohol Clin Exp Res (Hoboken). 2026 Jan 12;50(1):e70232. doi: 10.1111/acer.70232 (PMC12796562; doi:10.1111/acer.70232)
Supplement: Supplementary file 1 — Tables S1–S5 [file ACER-50-0-s001.docx]

Working memory in context: The role of alcohol distractors

in working memory performance in low to heavy alcohol drinkers

Karis Colyer-Patel^1,2*^, Emese Kroon^1^, Christophe Romein^1^, Hanan El Marroun^3,4^ Janna Cousijn^1^

^1^ Neuroscience of Addiction (NofA) Lab, Center for Substance use and Addiction Research (CESAR), Department of Psychology, Education & Child Studies, Erasmus University Rotterdam, Rotterdam, The Netherlands

^2^ University Medical Center Utrecht, UMC Brain Center, Department of Psychiatry, Utrecht, The Netherlands

^3^Department of Child and Adolescent Psychiatry/Psychology, Erasmus MC, University Medical Center Rotterdam, Rotterdam, the Netherlands.

^4^Department of Psychology, Education & Child Studies, Erasmus School of Social and Behavioural Sciences, Erasmus University Rotterdam, The Netherlands

### **Table of Contents**

Page 2: **Supplementary 1.** Full list of alcohol-related and neutral flanker words

Page 3: **Table S1.** Model selection and comparison for accuracy and reaction time.

Page 6: **Table S2.** Model selection and comparison for reaction time.

Page 10: **Table S3.** Model selection and comparison for accuracy.

Page 11: **Table S4.** Model selection and comparison for reaction time.

Page 13: **Supplementary Figure S1.** Mean reaction time across working memory load and flanker conditions for Block 1 and 2.

Page 14: **Table S5.** Internal consistency (Cronbach’s alpha) of mean reaction times across task blocks and n-back load conditions.

**Supplementary 1.** Full list of alcohol-related and neutral flanker words used in the Dutch and English version of the Alcohol flanker N-back task.

Alcohol related flanker words:

*English*

Vodka, Alcohol, Bacardi, Bar, Beer, Wine, Heineken, Rum, Whisky, Pub, Cocktail, Spirits, Lager, Tipsy

*Dutch*

Wodka, Alcohol, Bacardi, Bar, Bier, Wijn, Heineken, Rum, Whisky, Kroeg, Dronken, Zuipen, Pilsje, Shotje

Neutral related flanker words:

*English*

Pen, Computer, Lamp, Copy, Label, Paper, Notebook, Laptop, Glue, Fax, Pencil, Typing, Printing, Table.

*Dutch*

Pen, Computer, Lamp, Kopie, Etiket, Papier, Kladblok, Bureau, Lijm, Fax, Potlood, Typen, Printen, Tafel.

*Note*: the English and Dutch Flankers are not identical.

| **Table S1.** Model selection and comparison for accuracy. | | | | | | | | | | | |
| --- | --- | --- | --- | --- | --- | --- | --- | --- | --- | --- | --- |
| **Model** | | **Model coefficients** | | | | | | **Model comparison** | | | |
|  |  | **Fixed effects** | | | | | **Random effects** |  |  |  |  |
| **Accuracy** | | **B** | **95% CI (B)** | **SE (B)** | **t** | **p*** | **SD** | **AIC** | **BIC** | **X2** | **p** |
| 0 | (Intercept) | 88.39 | 87.75 : 89.03 | .33 | 271.53 | <.001 | 3.80 | 16767.90 | 16796.76 | - | - |
|  | WM-load | - | - | - | - | - | - |  |  |  |  |
|  | Flanker Type: Alcohol | - | - | - | - | - | - |  |  |  |  |
| 1 | (Intercept) | 94.91 | 94.08 : 95.73 | .42 | 225.49 | <.001 | 5.29 | 15902.26 | 15948.44 | 871.64 | <.001 |
|  | WM-load: 1-back | -3.18 | -4.00 : -2.36 | .42 | -7.60 | <.001 | 3.57 |  |  |  |  |
|  | WM-load: 2-back | -8.12 | -8.95 : -7.30 | .42 | -19.35 | <.001 |  |  |  |  |  |
|  | WM-load: 3-back | -15.20 | -16.02 : -14.37 | .42 | -36.15 | <.001 |  |  |  |  |  |
|  | Flanker type: Alcohol | - | - | - | - | - | 4.83 |  |  |  |  |
| 2 | (Intercept) | 95.00 | 94.14 : 95.84 | .43 | 218.75 | <.001 | 5.29 | 15903.68 | 15955.63 | 0.58 | .44 |
|  | WM-load: 1-back | -3.18 | -4.00 : -2.36 | .42 | -7.60 | <.001 | 3.57 |  |  |  |  |
|  | WM-load: 2-back | -8.12 | -8.95 : -7.30 | .42 | -19.35 | <.001 |  |  |  |  |  |
|  | WM-load: 3-back | -15.20 | -16.02 : -14.37 | .42 | -36.14 | <.001 |  |  |  |  |  |
|  | Flanker type: Alcohol | -0.17 | -0.59 : 0.26 | .22 | -0.76 | 1.0 | 4.83 |  |  |  |  |
| 3 | (Intercept) | 94.06 | 93.14 : 94.98 | 0.47 | 200.75 | <.001 | 5.29 | **15849.59** | **15918.87** | **60.08** | **<.001** |
|  | WM-load: 1-back | -2.75 | -3.75 : - 1.75 | 0.51 | -5.38 | <.001 | 3.69 |  |  |  |  |
|  | WM-load: 2-back | -5.99 | -6.99 : -4.98 | 0.51 | -11.68 | <.001 |  |  |  |  |  |
|  | WM-load: 3-back | -13.98 | -14.99 : -12.97 | 0.52 | -27.14 | <.001 |  |  |  |  |  |
|  | Flanker type: Alcohol | 1.73 | 0.92 : 2.54 | 0.42 | 4.16 | .002 | 4.68 |  |  |  |  |
|  | WM-load 1-back: Flanker Alcohol | -0.89 | -2.05 : 0.26 | 0.59 | -1.51 | 1.0 | - |  |  |  |  |
|  | WM-load 2-back: Flanker Alcohol | -4.34 | -5.51 : -3.18 | 0.59 | -7.31 | <.001 | - |  |  |  |  |
|  | WM-load 3-back: Flanker Alcohol | -2.47 | -3.63 : -1.30 | 0.60 | -4.15 | .002 |  |  |  |  |  |
| 4 | (Intercept) | 93.95 | 92.89 : 95.02 | 0.55 | 172.21 | <.001 | 5.25 | 15849.51 | 15930.33 | 4.08 | .13 |
|  | WM-load: 1-back | -2.75 | -3.75 : -1.75 | 0.51 | -5.38 | <.001 | 3.69 |  |  |  |  |
|  | WM-load: 2-back | -5.99 | -6.99 : -4.98 | 0.51 | -11.67 | <.001 |  |  |  |  |  |
|  | WM-load: 3-back | -13.98 | -14.99 : -12.97 | 0.51 | -27.13 | <.001 |  |  |  |  |  |
|  | Flanker type: Alcohol | 1.73 | 0.92 : 2.55 | 0.42 | 4.16 | .002 | 4.68 |  |  |  |  |
|  | Group High Risk | 1.91 | -0.20 : 4.01 | 1.07 | 1. 77 | 1.0 | - |  |  |  |  |
|  | Group Mid Risk | -0.38 | -1.86 : 1.09 | 0.75 | -0.51 | 1.0 | - |  |  |  |  |
|  | WM-load 1-back: Flanker Alcohol | -0.89 | -2.05 : 0.26 | 0.59 | -1.51 | 1.0 | - |  |  |  |  |
|  | WM-load 2-back: Flanker Alcohol | -4.34 | -5.51 : -3.18 | 0.59 | -7.31 | <.001 | - |  |  |  |  |
|  | WM-load 3-back: Flanker Alcohol | -2.47 | -3.64 : -1.31 | 0.60 | -4.15 | .002 | - |  |  |  |  |
| 5 | (Intercept) | 94.08 | 92.93 : 95.22 | 0.59 | 160.57 | <.001 | 5.26 | 15851.89 | 15967.35 | 9.62 | 0.14 |
|  | WM-load: 1-back | -3.39 | -4.60 : -2.17 | 0.62 | -5.44 | <.001 | 3.65 |  |  |  |  |
|  | WM-load: 2-back | -5.88 | -7.10 : -4.66 | 0.62 | -9.43 | <.001 |  |  |  |  |  |
|  | WM-load: 3-back | -13.96 | -15.18 : -12.74 | 0.63 | -22.31 | <.001 |  |  |  |  |  |
|  | Flanker type: Alcohol | 1.73 | 0.92 : 2.55 | 0.42 | 4.16 | .002 | 4.68 |  |  |  |  |
|  | Group High Risk | 01.22 | -1.42 : 3.87 | 1.35 | 0.91 | 1.0 | - |  |  |  |  |
|  | Group Mid Risk | -0.54 | -2.39 : 1.31 | 0.94 | -0.57 | 1.0 | - |  |  |  |  |
|  | WM-load 1-back: Flanker Alcohol | -0.88 | -2.04 : 0.27 | 0.59 | -1.50 | 1.0 | - |  |  |  |  |
|  | WM-load 2-back: Flanker Alcohol | -4.34 | -5.50 : -3.18 | 0.59 | -7.30 | <.001 | - |  |  |  |  |
|  | WM-load 3-back: Flanker Alcohol | -2.48 | -3.64 : -1.31 | 0.60 | -4.15 | .002 | - |  |  |  |  |
|  | WM-load 1-back: Group High Risk | 0.85 | -1.76 : 3.46 | 1.34 | 0.64 | 1.0 | - |  |  |  |  |
|  | WM-load 2-back: Group High Risk | 1.04 | -1.58 : 3.67 | 1.34 | 0.78 | 1.0 | - |  |  |  |  |
|  | WM-load 3-back: Group High Risk | 0.84 | -1.79 : 3.46 | 1.34 | 0.62 | 1.0 | - |  |  |  |  |
|  | WM-load 1-back: Group Mid Risk | 1.76 | -0.07 : 3.60 | 0.94 | 1.88 | 1.0 | - |  |  |  |  |
|  | WM-load 2-back: Group Mid Risk | -0.74 | -2.58 : 1.09 | 0.94 | -0.79 | 1.0 | - |  |  |  |  |
|  | WM-load 3-back: Group Mid Risk | -0.38 | -2.22 : 1.47 | 0.94 | -0.40 | 1.0 | - |  |  |  |  |
| 6 | (Intercept) | 94.00 | 92.84 : 95.16 | 0.59 | 158.66 | <.001 | 5.26 | 15854.32 | 15981.32 | 1.57 | 0.46 |
|  | WM-load: 1-back | -3.39 | -4.60 : -2.17 | 0.62 | -5.44 | <.001 | 3.65 |  |  |  |  |
|  | WM-load: 2-back | -5.88 | -7.10 : -4.66 | 0.62 | -9.43 | <.001 |  |  |  |  |  |
|  | WM-load: 3-back | -13.96 | -15.19 : -12.74 | 0.63 | -22.31 | <.001 |  |  |  |  |  |
|  | Flanker type: Alcohol | 1.89 | 1.01 : 2.78 | 0.45 | 4.17 | .002 | 4.68 |  |  |  |  |
|  | Group High Risk | 1.18 | -1.55 : 3.90 | 1.39 | 0.84 | 1.0 | - |  |  |  |  |
|  | Group Mid Risk | -0.27 | -2.17 : 1.64 | 0.97 | -0.28 | 1.0 | - |  |  |  |  |
|  | WM-load 1-back: Flanker Alcohol | -0.89 | -2.05 : 0.26 | 0.59 | -1.51 | 1.0 | - |  |  |  |  |
|  | WM-load 2-back: Flanker Alcohol | -4.34 | -5.51 : -3.18 | 0.59 | -7.30 | <.001 | - |  |  |  |  |
|  | WM-load 3-back: Flanker Alcohol | -2.48 | -3.64 : -1.31 | 0.60 | -4.16 | .002 | - |  |  |  |  |
|  | WM-load 1-back: Group High Risk | 0.85 | -1.76 : 3.47 | 1.34 | 0.64 | 0.46 | - |  |  |  |  |
|  | WM-load 2-back: Group High Risk | 1.05 | -1.58 : 3.67 | 1.34 | 0.78 | 1.0 | - |  |  |  |  |
|  | WM-load 3-back: Group High Risk | 0.84 | -1.79 : 3.46 | 1.34 | 0.62 | 1.0 | - |  |  |  |  |
|  | WM-load 1-back: Group Mid Risk | 1.76 | -0.08 : 3.60 | 0.94 | 1.87 | 1.0 | - |  |  |  |  |
|  | WM-load 2-back: Group Mid Risk | -0.74 | -2.58 : 1.09 | 0.94 | -0.79 | 1.0 | - |  |  |  |  |
|  | WM-load 3-back: Group Mid Risk | -0.38 | -2.22 : 1.46 | 0.94 | -0.40 | 1.0 | - |  |  |  |  |
|  | Flanker Alcohol: Group High Risk | 0.09 | -1.22 : 1.40 | 0.67 | 0.14 | 1.0 | - |  |  |  |  |
|  | Flanker Alcohol: Group Mid Risk | -0.55 | -1.48 : 0.37 | 0.47 | -1.17 | 1.0 | - |  |  |  |  |
| 7 | (Intercept) | 93.90 | 92.71 : 95.09 | 0.61 | 153.66 | <.001 | 5.26 | 15852.28 | 16013.91 | 14.05 | .029 |
|  | WM-load: 1-back | -2.90 | -4.21 : -1.59 | 0.67 | -4.33 | <.001 | 3.68 |  |  |  |  |
|  | WM-load: 2-back | -5.78 | -7.09 : -4.47 | 0.67 | -8.60 | <.001 |  |  |  |  |  |
|  | WM-load: 3-back | -14.15 | -15.46 : -12.83 | 0.67 | -20.98 | <.001 |  |  |  |  |  |
|  | Flanker type: Alcohol | 2.11 | 1.04 : 3.17 | 0.54 | 3.87 | .005 | 4.64 |  |  |  |  |
|  | Group High Risk | 1.47 | -1.48 : 4.43 | 1.51 | 0.98 | 1.0 | - |  |  |  |  |
|  | Group Mid Risk | -0.03 | -2.09 : 2.02 | 1.05 | -0.03 | 1.0 | - |  |  |  |  |
|  | WM-load 1-back: Flanker Alcohol | -1.87 | -3.37 : -0.36 | 0.77 | -2.42 | .65 | - |  |  |  |  |
|  | WM-load 2-back: Flanker Alcohol | -4.57 | -6.09 : -3.05 | 0.78 | -5.87 | <.001 | - |  |  |  |  |
|  | WM-load 3-back: Flanker Alcohol | -2.13 | -3.65 : -0.60 | 0.78 | -2.73 | .28 | - |  |  |  |  |
|  | WM-load 1-back: Group High Risk | -0.19 | -3.38 : 3.00 | 1.63 | -0.11 | 1.0 | - |  |  |  |  |
|  | WM-load 2-back: Group High Risk | 1.56 | -1.65 : 4.77 | 1.64 | 0.95 | 1.0 | - |  |  |  |  |
|  | WM-load 3-back: Group High Risk | 0.17 | -3.06 : 3.40 | 1.65 | 0.10 | 1.0 | - |  |  |  |  |
|  | WM-load 1-back: Group Mid Risk | 0.56 | -1.68 : 2.80 | 1.15 | 0.49 | 1.0 | - |  |  |  |  |
|  | WM-load 2-back: Group Mid Risk | -1.31 | -3.55 : 0.93 | 1.15 | -1.14 | 1.0 | - |  |  |  |  |
|  | WM-load 3-back: Group Mid Risk | 0.46 | -1.79 : 2.71 | 1.15 | 0.40 | 1.0 | - |  |  |  |  |
|  | Flanker Alcohol: Group High Risk | -1.03 | -3.12 : 2.10 | 1.34 | -0.38 | 1.0 | - |  |  |  |  |
|  | Flanker Alcohol: Group Mid Risk | -0.95 | -2.84 : 0.78 | 0.93 | -1.11 | 1.0 | - |  |  |  |  |
|  | WM-load 1-back: Flanker Alcohol:  Group High Risk | 2.09 | -1.57 : 5.75 | 1.87 | 1.11 | 1.0 | - |  |  |  |  |
|  | WM-load 2-back: Flanker Alcohol:  Group High Risk | -1.01 | -4.71 : 2.68 | 1.89 | -0.53 | 1.0 | - |  |  |  |  |
|  | WM-load 3-back: Flanker Alcohol:  Group High Risk | 1.29 | -2.41 : 4.98 | 1.89 | 0.68 | 1.0 | - |  |  |  |  |
|  | WM-load 1-back: Flanker Alcohol:  Group Mid Risk | 2.43 | -0.14 : 5.01 | 1.32 | 1.84 | 1.0 | - |  |  |  |  |
|  | WM-load 2-back: Flanker Alcohol:  Group Mid Risk | 1.14 | -1.45 : 3.72 | 1.32 | 0.86 | 1.0 | - |  |  |  |  |
|  | WM-load 3-back: Flanker Alcohol:  Group Mid Risk | -1.69 | -4.29 : 0.90 | 1.33 | -1.27 | 1.0 | - |  |  |  |  |

*Note.* Mixed model results using random intercept and maximum likelihood estimation. CI; confidence interval, SE; standard error, SD; standard deviation, WM; working memory * Holm corrected p-values are displayed. The final model was selected using a stepwise, nested model comparison approach in which predictors were added sequentially. Model 0 included only random intercepts (baseline). Model 1 added the main effect of WM-load. Model 2 additionally included flanker type, and Model 3 added the WM-load x flanker type interaction. Model 4 further included group, followed by the addition of the WM-load x Group interaction in Model 5 and the flanker type x group interaction in Model 6. Finally, Model 7 included the three way interaction between WM-load, flanker type and group.

| **Table S2.** Model selection and comparison for reaction time. | | | | | | | | | | | |
| --- | --- | --- | --- | --- | --- | --- | --- | --- | --- | --- | --- |
| **Model** | | **Model coefficients** | | | | | | **Model comparison** | | | |
|  |  | **Fixed effects** | | | | | **Random effects** |  |  |  |  |
| **Reaction Time** | | **B** | **95% CI (B)** | **SE (B)** | **t** | **p*** | **SD** | **AIC** | **BIC** | **X2** | **p** |
| 0 | (Intercept) | 782.03 | 767.70 : 796.35 | 7.30 | 107.10 | <.001 | 101.18 | 31248.16 | 31277.18 | - | - |
|  | WM-load | - | - | - | - | - | - |  |  |  |  |
|  | Flanker Type | - | - | - | - | - | - |  |  |  |  |
| 1 | (Intercept) | 632.21 | 614.83 : 649.58 | 8.86 | 71.32 | <.001 | 118.13 | 30444.46 | 30490.88 | 809.70 | < .001 |
|  | WM-load: 1-back | 105.52 | 89.43 : 122.60 | 8.20 | 12.87 | <.001 | 83.79 |  |  |  |  |
|  | WM-load: 2-back | 243.20 | 227.12 : 259.27 | 8.20 | 29.66 | <.001 |  |  |  |  |  |
|  | WM-load: 3-back | 252.22 | 236.12 : 268.32 | 8.21 | 30.72 | <.001 |  |  |  |  |  |
|  | Flanker type | - | - | - | - | - | - |  |  |  |  |
| 2 | (Intercept) | 625.32 | 607.65 : 642.99 | 9.01 | 69.37 | <.001 | 118.12 | 30428.70 | 30480.92 | 17.76 | < .001 |
|  | WM-load: 1-back | 105.43 | 89.35 : 121.51 | 8.20 | 12.85 | <.001 | 84.09 |  |  |  |  |
|  | WM-load: 2-back | 243.13 | 227.06 : 259.21 | 8.20 | 29.65 | <.001 |  |  |  |  |  |
|  | WM-load: 3-back | 252.17 | 236.07 : 268.27 | 8.21 | 30.71 | <.001 |  |  |  |  |  |
|  | Flanker type: Alcohol | 13.85 | 7.42 : 20.27 | 3.28 | 4.23 | .001 | 72.27 |  |  |  |  |
| 3 | (Intercept) | 645.82 | 627.42 : 664.23 | 9.40 | 68.73 | <.001 | 118.19 | **30317.95** | **30387.58** | **116.75** | **< .001** |
|  | WM-load: 1-back | 61.07 | 43.81 : 79.33 | 9.32 | 6.55 | <.001 | 85.78 |  |  |  |  |
|  | WM-load: 2-back | 234.21 | 215.96 : 252.46 | 9.31 | 25.14 | <.001 |  |  |  |  |  |
|  | WM-load: 3-back | 222.98 | 204.71 : 241.25 | 9.32 | 23.92 | <.001 |  |  |  |  |  |
|  | Flanker type: Alcohol | -27.37 | -39.57 : -15.18 | 6.23 | -4.40 | <.001 | 68.53 |  |  |  |  |
|  | WM-load 1-back: Flanker Alcohol | 88.55 | 71.26 : 105.84 | 8.83 | 10.03 | <.001 | - |  |  |  |  |
|  | WM-load 2-back: Flanker Alcohol | 18.24 | 0.93 : 35.55 | 8.84 | 2.06 | 1.0 | - |  |  |  |  |
|  | WM-load 3-back: Flanker Alcohol | 58.46 | 41.16 : 75.76 | 8.83 | 6.62 | <.001 |  |  |  |  |  |
| 4 | (Intercept) | 650.07 | 628.05 : 672.09 | 11.25 | 57.80 | <.001 | 118.08 | 30321.47 | 30402.70 | 0.49 | .78 |
|  | WM-load: 1-back | 61.07 | 42.81 : 79.32 | 9.32 | 6.55 | <.001 | 85.78 |  |  |  |  |
|  | WM-load: 2-back | 234.22 | 215.96 : 252.47 | 9.32 | 25.13 | <.001 |  |  |  |  |  |
|  | WM-load: 3-back | 222.98 | 204.71 : 241.25 | 9.33 | 23.91 | <.001 |  |  |  |  |  |
|  | Flanker type: Alcohol | -27.37 | -39.57 : -15.18 | 6.23 | -4.39 | <.001 | 68.53 |  |  |  |  |
|  | Group High Risk | -12.23 | -58.43 : 33.98 | 22.53 | -0.52 | 1.0 | - |  |  |  |  |
|  | Group Mid Risk | -9.41 | -41.70 : 22.88 | 16.44 | -0.57 | 1.0 | - |  |  |  |  |
|  | WM-load 1-back: Flanker Alcohol | 88.55 | 70.26 : 105.84 | 8.83 | 10.03 | <.001 | - |  |  |  |  |
|  | WM-load 2-back: Flanker Alcohol | 18.25 | 0.94: 35.55 | 8.84 | 2.06 | 1.0 | - |  |  |  |  |
|  | WM-load 3-back: Flanker Alcohol | 58.47 | 41.16 : 75.77 | 8.84 | 6.62 | <.001 | - |  |  |  |  |
| 5 | (Intercept) | 643.39 | 619.90 : 666.88 | 12.01 | 53.56 | <.001 | 118.22 | 30321.80 | 30437.85 | 11.67 | .07 |
|  | WM-load: 1-back | 69.40 | 46.76 : 92.04 | 11.57 | 6.00 | <.001 | 85.02 |  |  |  |  |
|  | WM-load: 2-back | 243.20 | 220.54 : 265.85 | 11.58 | 21.00 | <.001 |  |  |  |  |  |
|  | WM-load: 3-back | 232.50 | 209.86 : 255.15 | 11.58 | 20.08 | <.001 |  |  |  |  |  |
|  | Flanker type: Alcohol | -27.37 | -39.57 : -15.18 | 6.24 | -4.39 | <.001 | 68.56 |  |  |  |  |
|  | Group High Risk | -27.37 | -79.33 : 32.46 | 28.50 | -0.82 | 1.0 | - |  |  |  |  |
|  | Group Mid Risk | 17.17 | -21.88 : 56.22 | 19.91 | 0.86 | 1.0 | - |  |  |  |  |
|  | WM-load 1-back: Flanker Alcohol | 88.54 | 71.25 : 105.83 | 8.84 | 10.01 | <.001 | - |  |  |  |  |
|  | WM-load 2-back: Flanker Alcohol | 18.21 | 0.91 : 35.52 | 8.85 | 2.06 | 1.0 | - |  |  |  |  |
|  | WM-load 3-back: Flanker Alcohol | 58.46 | 41.16 : 75.76 | 8.85 | 6.61 | <.001 | - |  |  |  |  |
|  | WM-load 1-back: Group High Risk | -8.40 | -60.02 : 43.21 | 26.39 | -0.32 | 1.0 | - |  |  |  |  |
|  | WM-load 2-back: Group High Risk | 26.16 | -25.14 : 77.47 | 26.23 | 1.00 | 1.0 | - |  |  |  |  |
|  | WM-load 3-back: Group High Risk | 26.49 | -24.83 : 77.81 | 26.23 | 1.01 | 1.0 | - |  |  |  |  |
|  | WM-load 1-back: Group Mid Risk | -24.66 | -60.49 : 11.18 | 18.32 | -1.35 | 1.0 | - |  |  |  |  |
|  | WM-load 2-back: Group Mid Risk | -39.88 | -75.70 : -4.06 | 18.31 | -2.18 | 1.0 | - |  |  |  |  |
|  | WM-load 3-back: Group Mid Risk | -42.01 | -77.93 : -6.10 | 18.36 | -2.29 | .872 | - |  |  |  |  |
| 6 | (Intercept) | 643.85 | 620.22 : 667.48 | 12.09 | 53.26 | <.001 | 118.22 | 30325.66 | 30453.31 | .14 | .93 |
|  | WM-load: 1-back | 69.40 | 46.77 : 92.04 | 11.58 | 5.99 | <.001 | 85.03 |  |  |  |  |
|  | WM-load: 2-back | 243.21 | 220.56 : 265.86 | 11.59 | 20.99 | <.001 |  |  |  |  |  |
|  | WM-load: 3-back | 232.50 | 209.86 : 255.15 | 11.58 | 20.08 | <.001 |  |  |  |  |  |
|  | Flanker type: Alcohol | -28.30 | -41.54 : -15.06 | 6.77 | -4.18 | .001 | 68.56 |  |  |  |  |
|  | Group High Risk | -25.02 | -81.76 : 31.71 | 28.94 | -0.86 | 1.0 | - |  |  |  |  |
|  | Group Mid Risk | 16.25 | -23.40: 55.89 | 20.22 | 0.80 | 1.0 | - |  |  |  |  |
|  | WM-load 1-back: Flanker Alcohol | 88.54 | 71.25 : 105.83 | 8.84 | 10.01 | <.001 | - |  |  |  |  |
|  | WM-load 2-back: Flanker Alcohol | 18.20 | 0.89 : 35.50 | 8.85 | 2.06 | 1.0 | - |  |  |  |  |
|  | WM-load 3-back: Flanker Alcohol | 58.46 | 41.16 : 75.76 | 8.85 | 6.60 | <.001 | - |  |  |  |  |
|  | WM-load 1-back: Group High Risk | -8.44 | -60.05 : 43.18 | 26.40 | -0.32 | 1.0 | - |  |  |  |  |
|  | WM-load 2-back: Group High Risk | 26.11 | -25.20 : 77.42 | 26.24 | 1.00 | 1.0 | - |  |  |  |  |
|  | WM-load 3-back: Group High Risk | 26.44 | -24.88 : 77.76 | 26.25 | 1.01 | 1.0 | - |  |  |  |  |
|  | WM-load 1-back: Group Mid Risk | -24.67 | -60.50 : 11.17 | 18.33 | -1.35 | 1.0 | - |  |  |  |  |
|  | WM-load 2-back: Group Mid Risk | -39.88 | -75.70 : -4.06 | 18.32 | -2.18 | 1.0 | - |  |  |  |  |
|  | WM-load 3-back: Group Mid Risk | -42.02 | -77.94 : -6.10 | 18.37 | -2.29 | .872 | - |  |  |  |  |
|  | Flanker Alcohol: Group High Risk | 1.85 | -16.48 : 15.58 | 10.09 | 0.32 | 1.0 | - |  |  |  |  |
|  | Flanker Alcohol: Group Mid Risk | 3.24 | -11.88 : 15.58 | 7.02 | 0.26 | 1.0 | - |  |  |  |  |
| 7 | (Intercept) | 641.03 | 616.99 : 665.06 | 12.31 | 52.07 | <.001 | 118.24 | 30326.52 | 30488.99 | 11.14 | .08 |
|  | WM-load: 1-back | 73.33 | 49.56 : 97.11 | 12.17 | 6.02 | <.001 | 85.17 |  |  |  |  |
|  | WM-load: 2-back | 247.15 | 223.35 : 270.94 | 12.19 | 20.28 | <.001 |  |  |  |  |  |
|  | WM-load: 3-back | 236.02 | 212.26 : 259.78 | 12.17 | 19.40 | <.001 |  |  |  |  |  |
|  | Flanker type: Alcohol | -22.65 | -38.53 : -6.78 | 8.13 | -2.79 | .222 | 68.21 |  |  |  |  |
|  | Group High Risk | -36.39 | -95.52 : 22.74 | 30.20 | -1.20 | 1.0 | - |  |  |  |  |
|  | Group Mid Risk | 30.01 | -11.34 : 71.36 | 21.12 | 1.42 | 1.0 | - |  |  |  |  |
|  | WM-load 1-back: Flanker Alcohol | 80.71 | 58.20 : 103.22 | 11.53 | 7.00 | <.001 | - |  |  |  |  |
|  | WM-load 2-back: Flanker Alcohol | 10.34 | -12.27 : 32.96 | 11.58 | 0.89 | 1.0 | - |  |  |  |  |
|  | WM-load 3-back: Flanker Alcohol | 51.42 | 28.88 : 73.96 | 11.55 | 4.45 | <.001 | - |  |  |  |  |
|  | WM-load 1-back: Group High Risk | 13.54 | -44.96 : 72.03 | 29.95 | 0.45 | 1.0 |  |  |  |  |  |
|  | WM-load 2-back: Group High Risk | 32.52 | -25.83 : 90.86 | 29.88 | 1.09 | 1.0 | - |  |  |  |  |
|  | WM-load 3-back: Group High Risk | 43.90 | -14.43: 102.23 | 29.87 | 1.47 | 1.0 | - |  |  |  |  |
|  | WM-load 1-back: Group Mid Risk | -46.15 | -86.85 : -5.45 | 20.84 | -2.21 | 1.0 | - |  |  |  |  |
|  | WM-load 2-back: Group Mid Risk | -55.39 | -96.05 : -14.72 | 20.82 | -2.66 | .318 | - |  |  |  |  |
|  | WM-load 3-back: Group Mid Risk | -60.42 | -101.21 : -19.64 | 20.88 | -2.89 | .164 | - |  |  |  |  |
|  | Flanker Alcohol: Group High Risk | 26.61 | -12.59 : 65.82 | 20.08 | 1.33 | 1.0 | - |  |  |  |  |
|  | Flanker Alcohol: Group Mid Risk | -25.87 | -53.11 : 1.38 | 13.96 | -1.85 | 1.0 | - |  |  |  |  |
|  | WM-load 1-back: Flanker Alcohol:  Group High Risk | -44.63 | -100.16 : 10.89 | 28.44 | -1.57 | 1.0 | - |  |  |  |  |
|  | WM-load 2-back: Flanker Alcohol:  Group High Risk | -13.64 | -69.17 : 41.89 | 28.45 | -0.48 | 1.0 | - |  |  |  |  |
|  | WM-load 3-back: Flanker Alcohol:  Group High Risk | -35.27 | -90.74 : 20.21 | 28.42 | -1.24 | 1.0 | - |  |  |  |  |
|  | WM-load 1-back: Flanker Alcohol:  Group Mid Risk | 43.11 | 4.48 : 81.74 | 19.79 | 2.18 | 1.0 | - |  |  |  |  |
|  | WM-load 2-back: Flanker Alcohol:  Group Mid Risk | 31.18 | -7.39 : 69.75 | 19.76 | 1.58 | 1.0 | - |  |  |  |  |
|  | WM-load 3-back: Flanker Alcohol:  Group Mid Risk | 36.92 | -1.74 : 75.58 | 19.80 | 1.86 | 1.0 | - |  |  |  |  |

*Note.* Mixed model results using random intercept and maximum likelihood estimation. CI; confidence interval, SE; standard error, SD; standard deviation, WM; working memory * Holm corrected p-values are displayed. The final model was selected using a stepwise, nested model comparison approach in which predictors were added sequentially. Model 0 included only random intercepts (baseline). Model 1 added the main effect of WM-load. Model 2 additionally included flanker type, and Model 3 added the WM-load x flanker type interaction. Model 4 further included group, followed by the addition of the WM-load x Group interaction in Model 5 and the flanker type x group interaction in Model 6. Finally, Model 7 included the three-way interaction between WM-load, flanker type and group.

| **Table S3.** Model selection and comparison for accuracy. | | | | | | | | | | | |
| --- | --- | --- | --- | --- | --- | --- | --- | --- | --- | --- | --- |
| **Model** | | **Model coefficients** | | | | | | **Model comparison** | | | |
|  |  | **Fixed effects** | | | | | **Random effects** |  |  |  |  |
| **Difference Score of accuracy**  **(3 back – 1 back)** | | **B** | **95% CI (B)** | **SE (B)** | **t** | **p** | **SD** | **AIC** | **BIC** | **X2** | **p** |
| 0 | (Intercept) | -11.97 | -12.87 : -11.07 | .46 | -26.07 | <.001 | 5.95 | 4153.48 | 4170.89 | - | - |
|  | Flanker Type | - | - | - | - | - | 6.69 |  |  |  |  |
| 1 | (Intercept) | -11.25 | -12.33 : -10.17 | .55 | -20.48 | <.001 | 6.01 | **4149.87** | **4171.64** | **5.61** | **.018** |
|  | Flanker type: Alcohol | -1.43 | -2.61 : -0.25 | .60 | -2.38 | .137 | 6.61 |  |  |  |  |
| 2 | (Intercept) | -10.64 | -11.97 : -9.32 | .67 | -15.78 | <.001 | 5.94 | 4150.02 | 4180.49 | 3.86 | .145 |
|  | Flanker type: Alcohol | -1.45 | -2.63 : -0.26 | .60 | -2.40 | .137 | 6.62 |  |  |  |  |
|  | Group Mid Risk | -1.97 | -3.98 : 0.05 | 1.03 | -1.91 | .340 | - |  |  |  |  |
|  | Group High Risk | -0.06 | -2.90 : 2.77 | 1.45 | 0.04 | 1.0 | - |  |  |  |  |
| 3 | (Intercept) | -11.31 | -12.72 : -9.90 | .72 | -15.71 | <.001 | 6.02 | 4144.53 | 4183.70 | 9.49 | .009 |
|  | Flanker type: Alcohol | -0.11 | -1.64 : 1.41 | .78 | -0.15 | 1.0 | 6.49 |  |  |  |  |
|  | Group Mid Risk | -0.05 | -2.34 : 2.44 | 1.22 | 0.04 | 1.0 | - |  |  |  |  |
|  | Group High Risk | 0.37 | -3.04 : 3.78 | 1.74 | .21 | 1.0 | - |  |  |  |  |
|  | Flanker Alcohol: Group Mid Risk | -4.12 | -6.74 : -1.50 | 1.34 | -3.08 | .021 | - |  |  |  |  |
|  | Flanker Alcohol : Group High Risk | -0.89 | -4.54 : 2.77 | 1.86 | -0.47 | 1.0 | - |  |  |  |  |

*Note.* Mixed model results using random intercept and maximum likelihood estimation. CI; confidence interval, SE; standard error, SD; standard deviation. * Holm corrected p-values are displayed. The final model was selected using a stepwise, nested model comparison approach in which predictors were added sequentially. Model 0 included only random intercepts (baseline). Model 1 added the main effect of Flanker. Model 2 additionally included group, and Model 3 added the group x flanker interaction.

| **Table S4.** Model selection and comparison for reaction time. | | | | | | | | | | | |
| --- | --- | --- | --- | --- | --- | --- | --- | --- | --- | --- | --- |
| **Model** | | **Model coefficients** | | | | | | **Model comparison** | | | |
|  |  | **Fixed effects** | | | | | **Random effects** |  |  |  |  |
| **Difference Score of Reaction Time**  **(3 back – 1 back)** | | **B** | **95% CI (B)** | **SE (B)** | **t** | **p** | **SD** | **AIC** | **BIC** | **X2** | **p** |
| 0 | (Intercept) | 146.00 | 127.74 : 164.26 | 9.29 | 15.72 | < .001 | 139.66 | 7844.66 | 7862.26 | - | - |
|  | Flanker Type | - | - | - | - | - | 104.55 |  |  |  |  |
| 1 | (Intercept) | 160.04 | 140.62 : 181.46 | 10.39 | 15.49 | < .001 | 140.42 | **7836.37** | **7858.37** | **10.11** | **.0015** |
|  | Flanker type: Alcohol | -29.93 | -48.15 : -11.72 | 9.27 | -3.23 | .012 | 102.63 |  |  |  |  |
| 2 | (Intercept) | 161.89 | 136.38 : 187.39 | 13.00 | 12.45 | < .001 | 139.71 | 7838.05 | 7868.85 | .74 | .69 |
|  | Flanker type: Alcohol | -29.98 | -48.19 : -11.77 | 9.29 | -3.23 | .012 | 102.67 |  |  |  |  |
|  | Group Mid Risk | -15.43 | -56.20 : 25.34 | 20.79 | -.74 | 1.0 | - |  |  |  |  |
|  | Group High Risk | 33.43 | -25.31: 92.16 | 29.95 | 1.12 | 1.0 | - |  |  |  |  |
| 3 | (Intercept) | 161.20 | 134.55 : 187.85 | 13.61 | 11.84 | < .001 | 139.76 | 7841.61 | 7881.21 | 1.97 | .37 |
|  | Flanker type: Alcohol | -28.62 | -52.46 : -4.78 | 12.17 | -2.35 | .136 | 102.58 |  |  |  |  |
|  | Group Mid Risk | -10.98 | -56.58 : 34.62 | 23.29 | -0.47 | 1.0 | - |  |  |  |  |
|  | Group High Risk | 27.49 | -38.47 : 93.44 | 32.69 | 0.82 | 1.0 | - |  |  |  |  |
|  | Flanker Alcohol: Group Mid Risk | -8.88 | -49.65: 31.89 | 20.82 | -0.43 | 1.0 | - |  |  |  |  |
|  | Flanker Alcohol : Group High Risk | 11.59 | -47.24 : 70.41 | 30.04 | 0.39 | 1.0 | - |  |  |  |  |

*Note.* Mixed model results using random intercept and maximum likelihood estimation. CI; confidence interval, SE; standard error, SD; standard deviation. * Holm corrected p-values are displayed. Model 0 included only random intercepts (baseline). Model 1 added the main effect of Flanker. Model 2 additionally included group, and Model 3 added the group x flanker interaction.

**Supplementary Figure S1.** Mean reaction time across working memory load and flanker conditions for Block 1 and 2.


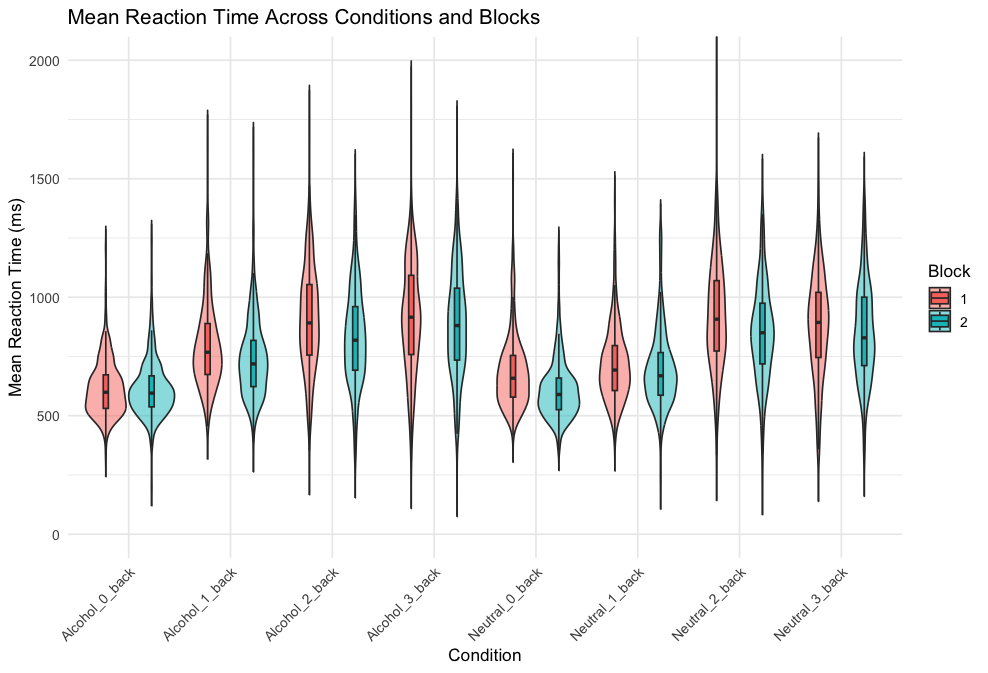


*Note.* Violin plot shows the distribution of participant level mean reaction times (ms) across working memory load (0-back, 1-back, 2-back, 3-back) and flanker conditions (Alcohol vs neutral), presented separately for Block 1 and Block 2. Mean reaction times were calculated from correct trials only, with implausible reaction times (<200ms) removed.

**Table S5.** Internal consistency (Cronbach’s alpha) of mean reaction times across task blocks and n-back load conditions.

| **Description** | **Cronbach’s α** |
| --- | --- |
| All conditions per block | |
| Block 1 | 0.84 |
| Block 2 | 0.84 |
| Across load per block | |
| Block 1 0-back | 0.68 |
| Block 2 0-back | 0.78 |
| Block 1 1-back | 0.72 |
| Block 2 1-back | 0.72 |
| Block 1 2-back | 0.69 |
| Block 2 2-back | 0.80 |
| Block 1 3-back | 0.74 |
| Block 2 3-back | 0.77 |

*Note.* Cronbach’s α reflect internal consistency of mean reaction times across alcohol and neutral conditions within each 18-trial block and n-back load.
